# High Expression of *TACC3* Is Associated with the Poor Prognosis and Immune Infiltration in Lung Adenocarcinoma Patients

**DOI:** 10.1155/2022/8789515

**Published:** 2022-07-09

**Authors:** Yan Chen, Min Zhou, Xuyu Gu, Longfei Wang, Cailian Wang

**Affiliations:** ^1^Department of Oncology, Zhongda Hospital, School of Medicine, Southeast University, Nanjing 210009, China; ^2^Department of Pathology, Zhongda Hospital, Southeast University, Nanjing 210009, China

## Abstract

**Background:**

Lung adenocarcinoma (LUAD) has been recognized as one of the commonest aggressive malignant tumors occurring in humans. The transforming acidic coiled-coil-containing protein 3 (*TACC3*) seems to be a probable prognostic marker and treatment target for non-small-cell lung cancer (NSCLC). Nevertheless, there exist no reports on the association between *TACC3* and immunotherapy or other therapeutic interventions in LUAD.

**Methods:**

Premised on the data accessed from The Cancer Genome Atlas- (TCGA-) LUAD, we carried out bioinformatics analysis. The *TACC3* expression in LUAD was analyzed utilizing the GEPIA. A survival module was constructed to evaluate the effect of *TACC3* on the survival of patients with LUAD. Logistic regression was undertaken to examine the relationship between *TACC3* expression and clinical factors. Protein-protein interaction analysis was performed in the GeneMANIA database, and enrichment analysis and identification of predicted signaling pathways were performed using Gene Ontology and Kyoto Encyclopedia of Genes. Additionally, the Cox regression was used to assess the clinicopathologic features linked to the overall survival in TCGA patients. Lastly, we investigated the link between *TACC3* and tumor-infiltrating immune cells (TIICs) through CIBERSORT and the “Correlation” module of GEPIA. The association between TACC3 gene expression and drug response was analyzed using the CellMiner database to predict drug sensitivity.

**Results:**

The outcomes illustrated that *TACC3* was upregulated and considerably correlated with dismal prognosis in LUAD patients. Moreover, the multivariate Cox regression analysis depicted *TACC3* as an independent prognostic marker in LUAD patients. It was also revealed that the expression of *TACC3* was related to clinical stage (*P* = 0.014), age (*P* = 0.002), and T classification (*P* ≤ 0.018). Moreover, we discovered that the expression of *TACC3* was considerably linked to a wide range of TIICs, especially the T cells and NK cells. Single-cell results found that TACC3 was mainly expressed in the immune cells (especially tprolif cells) and malignant cells. TACC3 gene expression was positively correlated with TMB and MSI, and TACC3 may provide a prediction of the efficacy of immunotherapy. Moreover, the correlation analysis between TACC3 gene expression and immune checkpoint gene expression revealed that TACC3 may coordinate the activities of these ICP genes in different signal transduction pathways. TACC3 is related to biological progress (BP), cellular component (CC), and molecular function (MF). The pathways involved in the interaction network involving TACC3 include nonhomologous end-joining, RNA transport, pantothenate and CoA biosynthesis, homologous recombination, and nucleotide excision repair. Furthermore, we investigated the association between the expression of TACC3 and the use of antitumor drugs, and TACC3 was positively correlated with response to most drugs.

**Conclusion:**

The findings from this research offer robust proof that the expression of *TACC3* could be a prognostic marker correlated with TIICs in LUAD. TACC3 can also provide new ideas for immunotherapy as a potential therapeutic target.

## 1. Introduction

Premised on the global cancer epidemiology statistics in 2020, lung cancer is still the deadliest malignancy in the world, with a cumulative incidence every year [1]. Nearly 85 percent of lung cancer incidents are non-small-cell lung cancers (NSCLC), with lung adenocarcinoma (LUAD) being the commonest histological subcategory [2]. Thus, it is extremely significant to identify auspicious biomarkers which are candidly associated with the prognosis as well as targeting for LUAD immunotherapy. The tumor-induced immune suppression is linked to differentiation in the infiltration metastasis and epigenetics in the tumor microenvironment (TME). Tumor pathogenesis involves an exceedingly intricate pathological process of regulating the incessant dynamic interplay between tumor tissues and the constituents of the adjacent immune system through activating various signaling pathways, thus affecting the prognosis as well as the survival of patients with LUAD.

The transforming acidic coiled-coil protein (TACC) family is distinguished by a conserved C-terminal “TACC domain,” which is important in the interplay with tubulin and microtubules and is recognized as having a major function in regulating microtubule and centrosome dynamics [3]. Therefore, they have a vital function in regulating many cellular functions such as cell growth, apoptosis, development, and differentiation. Besides, lots of research showed that the TACC family participated in the process of EMT and could enhance some oncogene expression to facilitate cancer development [4, 5]. The 3 main TACC proteins found in humans include TACC1, TACC2, and TACC3 [6]. TACC3 is known to participate in assembling and organizing microtubules and aligning chromosomes in the process of mitosis, maintaining the structure of the nuclear envelope, and regulating gene transcription as well as cell growth/differentiation [7]. The exhaustion of TACC3 results in embryonic lethality and growth retardation in mice owing to the increase in apoptosis [8]. Overexpression of TACC3 at both the protein and mRNA levels has been observed in more than 40 types of cancers [9]. TACC3 was also found to be highly expressed in lung cancer [10]. Moreover, fibroblast growth factor receptor (FGFR)1-TACC1 and FGFR3-TACC3 gene fusions have been reported in various types of cancers [10, 11]. These fusion proteins are associated with aneuploidy and display oncogenic activity. These results illustrate that TACC3 could possibly lead to tumorigenesis. Besides, the elevated TACC3 expression level was linked to advanced clinicopathological categorizations as well as adverse overall survival (OS) and free survival (RFS) in patients with NSCLC [12]. It is suggested that TACC3 might be a probable prognostic indicator and treatment target for NSCLC [13]. However, its potential role in LUAD is yet to be verified. The aim of this research is to assess the prognostic potential of TACC3 and comprehensively analyze the correlations between TACC3 and immune infiltration in LUAD.

The TME has been found to be a complex system comprising of diverse secreted cytokines and diverse cells such as immune cells. Immune cells infiltrating into the TME have a significant function in tumor immunogenicity which influences the incidence and progression of tumors [14]. Up to date, their proportion and composition could be utilized in the prediction and prognosis of many cancers. Their activities, density, and the site of tumor regions appear to have crucial functions in immune surveillance and defense. The T lymphocyte abundance in the TME has a strong influence on cancer prognosis, and it is often correlated with the immune surveillance of cancer in humans [15, 16]. Several research reports have illustrated that in a subgroup of patients with NSCLC having an extensive percentage of CD8+ T cells, only the ones having a high density and tumor-infiltrating lymphocyte- (TIL-) associated dendritic cells (DCs) have substantial clinical advantages as opposed to patients having a lower DC density [17]. The quantity of tumor-infiltrating mature DCs could be utilized to detect the patients with early-stage NSCLC and exposed to a greater risk of relapse [18]. The analysis of immunocellular infiltration density in tumor areas offers a more inclusive comprehension of endogenous antitumor immunity.

**Figure 1:**
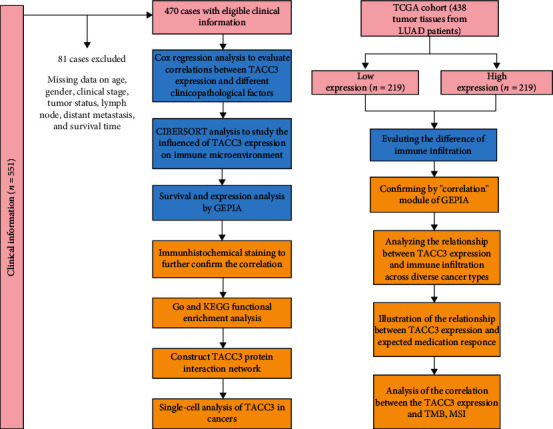
Study workflow.

Firstly, we analyzed the expression of *TACC3* as well as its prognostic value in LUAD patients. The correlation between the expression level of *TACC3* and the clinical features of patients with LUAD was assessed via an inclusive bioinformatics analysis of the gene expression profiles in LUAD. Subsequently, CIBERSORT which has been extensively acknowledged and the most utilized assessment algorithm was employed in analyzing the association between TACC3 and the relative abundance of TIICs in the TME [19]. Besides, we additionally approximated an OS analysis premised on the relative abundance of various TIIC subcategories. Then, GSEA was utilized to detect and rank the associated biological pathways to determine the possible mechanism of the candidate genes. The results of this research enhance our comprehension of the potential positive influence of TACC3 in LUAD and illuminate a potential correlation and probable mechanism between TACC3 and tumor-immune interplay. Thus, TACC3 appears to have the potential of becoming a new predictor that could assess the immune infiltration and prognosis in LUAD patients. This study's workflow is displayed in Figure 1.

## 2. Materials and Methods

### 2.1. Downloading and Preprocessing Data

The Cancer Genome Atlas (TCGA) database was utilized to extract the data on gene expression profiles of 551 patients with LUAD and 54 non-LUAD normal samples' clinical features of corresponding patients. The TCGA (https://TCGAData.nci.nih.gov/TCGA/) is a widely recognized open-access public directory that provides high-throughput microarray experimental information. The sequencing data of LUAD were produced utilizing the Illumina HiSeq_RNA-Seq forum. Subsequently, we processed the survival data of the TCGA-LUAD to screen out incidents with missing or insufficient information. Eventually, only 470 incidents that had eligible clinical data were considered for the Cox regression analysis. Then, the influence of *TACC3* on the constituent ratio of various immune cell subcategories in the 551 LUAD tumor tissues was determined utilizing the CIBERSORT. The preprocessing processes were carried out utilizing the Strawberry Perl and R (https://cran.r-project.org/) (R software, version 4.0).

### 2.2. Correlation between the Expression of *TACC3* and Survival Analysis of LUAD by GEPIA

The GEPIA webpage (http://GEPIA.cancerpku.cn/index.html) was utilized to analyze the expression of *TACC3* from the TCGA dataset [20]. The GEPIA is an extensively recognized public site that is utilized to process RNA sequencing data for an aggregate of 9736 tumor samples and 8587 nontumor normal specimens from the GTEx and TCGA. We merged GTEx and TCGA database data that can effectively solve the deficiency of insufficient normal tissue sample size in TCGA database, thereby improving the accuracy of comparison. The GEPIA was utilized to examine the link between the expression level of *TACC3* and clinicopathological factors. The link between the expression of *TACC3* and prognosis in LUAD patients was evaluated via the “survival” module of GEPIA. In the meantime, boxplot utilizing disease state (tumor or normal) as the variable was plotted to display differential *TACC3* expression between tumor and nontumor normal tissues. Additionally, clinical staging boxplot utilizing the pathological stage as the variable was plotted to contrast the expression of *TACC3* in various pathological stages.

### 2.3. Correlation of TACC3 with TMB and MSI

The unified and standardized pancancer dataset (TCGA Pancancer) was downloaded from the UCSC Xena database, and the expression of TACC3 (ENSG00000013810.18) gene in each sample was further extracted from it. In addition, we also used the TCGAmutations package to download the pancancer mutation data, calculated the TMB (tumor mutation burden) value of each tumor sample, integrated the TMB and TACC3 gene expression data of the sample, and used the R cor function to calculate the TMB and TACC3 in each tumor expression correlation.

### 2.4. Gene Set Enrichment Analysis (GSEA)

GSEA is a commonly utilized technique of computation that identifies whether an a *priori* specified gene sets depict statistically significant differential expression between low and high expression cohorts. 18 phenotype labels and dataset files were produced and input into the GSEA software. These phenotype labels comprised TACC3-low and TACC3-high. In each analysis, the permutations for the gene sets were conducted 1000 times. The gene sets that exhibited a normal false discovery rate (FDR) < 0.25 and a *P* value < 0.05 were deemed to be enriched.

### 2.5. Analysis of the Relative TIIC Abundance

CIBERSORT (http://cibersort.stanford.edu/) [21] is an extensively recognized online analytical site that is utilized to assess the significance of the relationship between TIICs and gene expression in tumors [22, 23]. It can be utilized in characterizing the cellular heterogeneity premised on the gene expression profiles of intricate tissues [24]. Moreover, the outcomes have been found to be extremely harmonious with the elementary truth estimates in different cancers [19]. Next, we employed the LM22-signed algorithm to the altered standard-annotated gene expression information. The exact process involved is described below. Premised on the gene annotation matrix of 22 immune cell subcategories accessed from the CIBERSORT webpage, we computed the *P* value of each specimen as per the deconvolution algorithm. The *P* < 0.05 was deemed to be statistically significant. The CIBERSORT is renowned for accurately and sensitively identifying the subtypes of immune cells. Since the inferred part of the relative abundance of immune cell subtype employed by CIBERSORT is precise, it could be analyzed further. Our grouping is based on the median value of *TACC3* in 438 LUAD patients categorized into low expression cohort (*n* = 219) and high cohort (*n* = 219). The CIBERSORT algorithm could detect the composition of the invasive immune cell for each specimen, thus allowing for effective comparison of the relative percentage of immune cells between the low and high expression cohorts of TACC3. Validation of tumor purity adjusted association of LAGE3 expression as well as the TILs abundance, such as B cells, dendritic cells, neutrophils, macrophages, CD4^+^ T cells, and CD8^+^ T cells in LUAD, was done utilizing the “gene” modules of TIMER [25]. The TIMER facilitates the systematic analysis of immune infiltrates among various types of diverse cancer (https://cistrome.shinyapps.io/ timer/).

### 2.6. Single-Cell Analysis of TACC3

Related single-cell analysis was applied by the Tumor Immune Single-Cell Hub (TISCH) web tool. The analysis parameters were as follows: TACC33 (gene), major lineage (cell-type annotation), and all cancers (cancer type). The expression levels of TACC3 in each cell type were quantified and visualized by a heatmap.

### 2.7. Construction of Protein Interaction Network

GeneMANIA (https://genemania.org/) uses extensive genomics and proteomic data to discover functionally similar genes. In this mode, it weights each functional genomics dataset according to the predicted value of the query. GeneMANIA was used to analyze TACC3 PPI in this investigation. At the same time, GO enrichment analysis was performed on the interaction genes obtained by using the R clusterProfiler package, and KEGG was performed on PPI genes with the help of the online tool Enrichr (https://maayanlab.cloud/Enrichr/). Relevant pathways are displayed as bar graphs.

### 2.8. Patients and Tissue Samples

The tissue specimens utilized in these experiments were obtained via surgical resection at the Zhongda Hospital Southeast University (Nanjing City, Jiangsu Province, China) between January 2013 and December 2015. Authorization for this research was obtained from the Ethics Committee of Zhongda Hospital Southeast University, and all enrolled patients gave a written informed consent (Ethics Number:2021ZDSYLL090-P01). 4% formalin was utilized to fix the tissue specimens for a duration of 24 hours. Next, these tissues were entrenched in paraffin to synthesize tissue chips in the Department of Pathology. These tissue chips which included 81 specimens of LUAD patients were diagnosed independently by 2 qualified pathologists. The clinicopathological features of the patients consisted of age, smoking history, sex, lymph node status, tumor size, differentiation, stage, and survival time (the period between surgery and death or end of follow-up).

### 2.9. Immunohistochemical (IHC) Staining

The paraffin mass of each tissue was cut into paraffin sections with a thickness of 5 *μ*m. The paraffin sections were routinely dewaxed. Immunohistochemistry was performed with En Vision two-step method and DAB. PBS buffer was utilized to substitute the primary antibody as the negative control, and known positive sections were used as the positive control. The specific staining procedures were carried out in strict adherence to the kit instructions. Both the test kit and the primary antibody *TACC3* were purchased from Beijing Solaibao Technology Co., Ltd. The positive staining of *TACC3* protein was located in the cytoplasm and nucleus, and the staining was light yellow, yellow, brownish yellow, and yellowish brown.

Under 200 times field of vision, three nonoverlapping fields were randomly selected from each slice, and Image ProPlus software was used for semiquantitative analysis of immunohistochemical results. The average optical density (IOD/area) = cumulative optical density/area of staining area measured. The larger the value, the stronger the positive. The criteria are as follows: 0-point, positive area < 2%; 1-point, positive area 2%~10%; 2 points, positive area accounted for 10%~20%; 3 points, positive area accounted for 21%~30%; and 4 points, positive area > 30%.

### 2.10. TACC3 Drug Sensitivity Prediction

The CellMiner database is primarily based on the 60 types of cancer cells listed by the National Cancer Institute's Center for Cancer Research (NCI). First, go to the CellMiner database homepage, click Download Data Sets, enter the data download interface, and select RNA expression data (RNA: RNA-seq) and drug data (Compound activity: DTP NCI-60); NCI-60 cell line is currently the most used cell line extensive sample population of cancer cells for anticancer drug testing. TACC3 gene expression data was extracted from the data, and the correlation coefficient between TACC3 and drugs was calculated with the help of the R cor function and visualized using R ggplot2.

### 2.11. Statistical Analysis

The link between clinical characteristics and *TACC3* expression was examined utilizing the logistic regression and Wilcoxon signed-rank test. The clinical features associated with the survival status of patients with LUAD were detected utilizing the Kaplan-Meier (KM) plot and Cox regression. The multivariate Cox analysis was employed to examine the function of *TACC3* expression in survival together with other clinical factors such as age, sex, smoking history, stage, differentiation, tumor size, and lymph node status. Low and high expression of *TACC3* was ascertained premised on the median values. Utilizing the median risk score of the expression of *TACC3* as the cutoff value, all the patients included in this research were categorized into high expression or low expression cohorts. The *P* value that was below 0.05 was deemed to be statistically significant. Spearman's R and statistical significance were utilized to assess the gene expression correlation. The R software (V.4.0.2) was utilized to carry out all the statistical analyses. Use glmnet package to implement logistic regression model. The OR value obtained from the model is shown in a forest plot. A logical regression model is implemented using GLMNET package. The OR value obtained from the model is shown in the forest diagram.

## 3. Results

### 3.1. Correlation between *TACC3* Expression and Clinical Features

**Figure 2:**
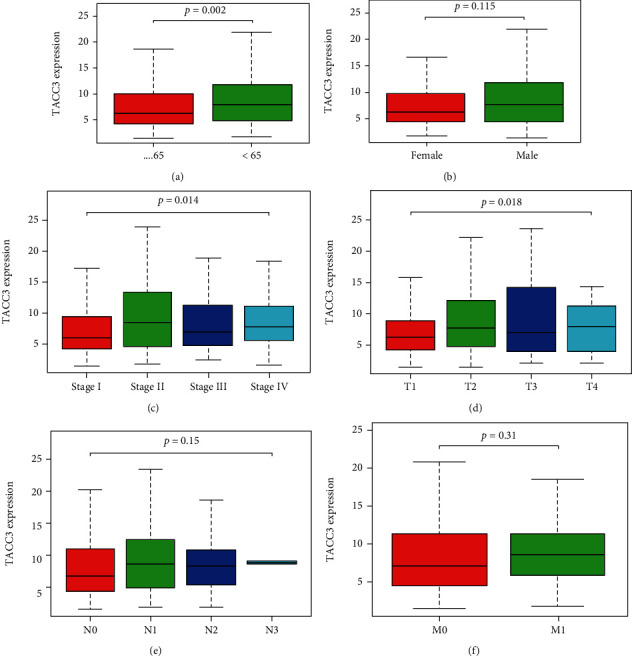
Association of *TACC3* expression with clinical variables. (a) Age. (b) Gender. (c) Stage. (d) Tumor stage. (e) Lymph node metastasis. (f) Distant metastases between *TACC3* expression and overall survival in LUAD patients in TCGA cohort.

**Figure 3:**
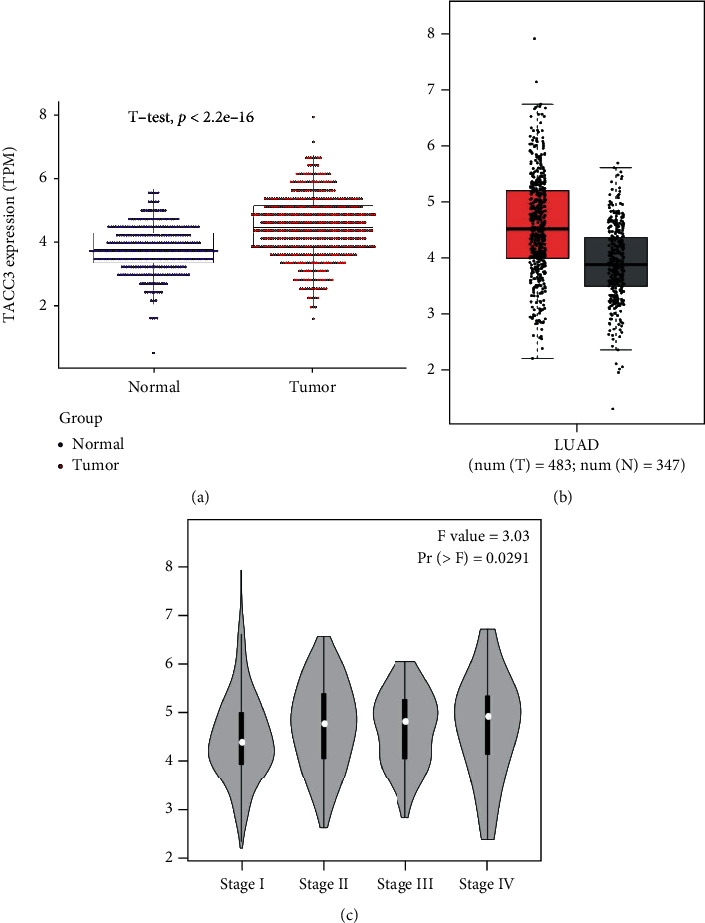
Associations between *TACC3* expression and clinical parameters. (a) Different expressions of *TACC3* in LUAD tissue and normal tissue. (b) Different expressions of *TACC3* from GEPIA analysis for verification. (c) Significant differences in *TACC3* expression in different pathological stages.

The analysis of the clinical information relating to the 551 LUAD patients from the TCGA was conducted. The information used in the analysis comprised of the patient's age, sex, lymph node, tumor status, distant metastasis, clinical stage, survival status, and survival time. As depicted in Figures 2(a)–2(f), the outcomes of contrasting the expression of *TACC3* in LUAD (515 samples) and non-LUAD normal tissues (347 samples) illustrated that the expression level of *TACC3* was elevated in LUAD (*P* < 0.001). The expression of *TACC3* in LUAD patients considerably increased as opposed to that in the non-LUAD normal cohort (*P* < 0.01) (Figure 3(a)). The same trend result was verified by GEPIA analysis (*P* < 0.01) (Figure 3(b)). The expression of *TACC3* was also considerably linked to the clinical stage (*P* = 0.014), age (*P* = 0.002), and T classification (*P* ≤ 0.018). By utilizing GEPIA, we discovered that the expression level of *TACC3* was considerably linked to the clinical stage (*P* = 0.0291) (Figure 3(c)). Univariate analysis premised on the logistic regression depicted that the expression level of *TACC3* was linked to undesirable prognostic clinicopathological factors (Table 1). The elevated expression of *TACC3* in LUAD was considerably linked to age (OR = 1.835 for age < 65 vs. age > 65), N classification (OR = 1.795 for N1 vs. N0), T classification (OR = 1.538 for T2 vs. T1), and high stage (OR = 1.912 for stage II vs. I). These results illustrated that patients having elevated *TACC3* expression levels seemed to proceed to a more advanced clinical stage as opposed to those having reduced *TACC3* expression.

### 3.2. Expression of *TACC3* Protein in Lung Adenocarcinoma and Adjacent Tissues

**Figure 4:**
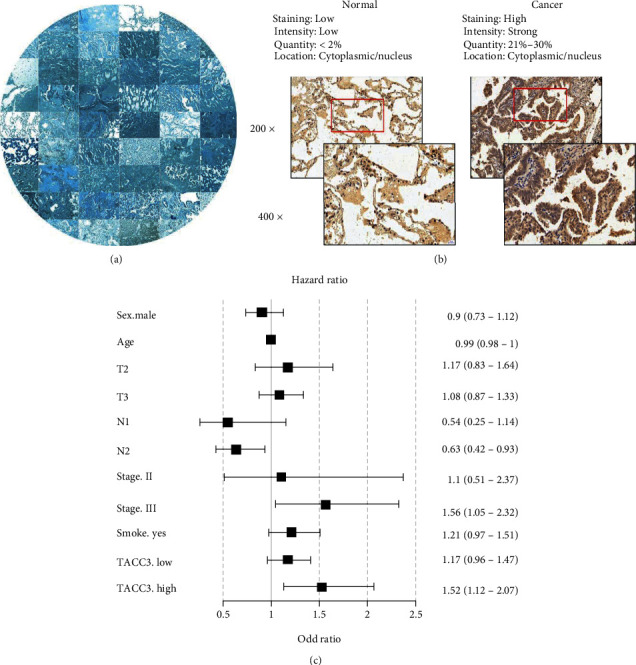
Validation of protein expression of *TACC3* protein in lung adenocarcinoma and adjacent tissues using immunohistochemical staining and correlation between *TACC3* and the prognosis. (a) Immunohistochemical staining results of *TACC3* in LUAD. (b) Expression of *TACC3* protein in lung adenocarcinoma and adjacent tissues. (c) Multivariate Cox analysis of *TACC3* expression and other clinicopathological features.

To further examine *TACC3* protein expression, we detected the expression of *TACC3* protein by immunohistochemical staining in LUAD and paracancerous tissues (Figure 4(a)). The level of *TACC3* expression in lung cancer tissues was highly elevated as opposed to that in adjoining nontumor normal tissues (Figure 4(b)). *TACC3* was expressed in the cytoplasm. The cancer cells grew multilayered along the alveolar wall, which was like an adenoid structure with papilla formation. The alveolar septum was not destroyed and the alveolar contour was preserved. Premised on the staining index, we categorized the samples into *TACC3*-high expression, *TACC3*-low expression, and *TACC3*-negative expression. As presented in Table 2, *TACC3* expression was associated with survival status (*P* = 0.013), while no link was found with age, smoking history, sex, M stage, N stage, T stage, or clinical stage. In the multivariate analysis, elevated *TACC3* expression (HR = 1.52, 95% CI = 1.12 − 2.07), age (HR = 0.99, 95% CI = 0.98 − 1), and N stage (HR = 0.63, 95% CI = 0.42 − 0.93) were independent prognostic predictors, as was depicted by the forest map (Figure 4(c)).

### 3.3. Diagnostic Value of *TACC3* Expression in LUAD

**Figure 5:**
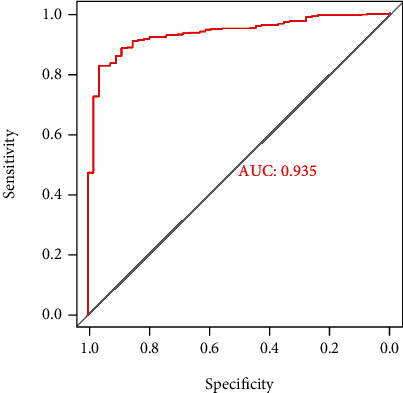
ROC curve for *TACC3* expression in LUAD tissue and normal tissue.

To assess TACC3's diagnostic value, we plotted a receiver operating characteristic (ROC) curve utilizing the expression data premised on 497 LUAD patients and 54 non-LUAD healthy persons. Analysis of the area under the ROC curve produced a value of 0.935, which indicated a considerable diagnostic value (Figure 5).

### 3.4. Survival and Multivariate Analysis

**Figure 6:**
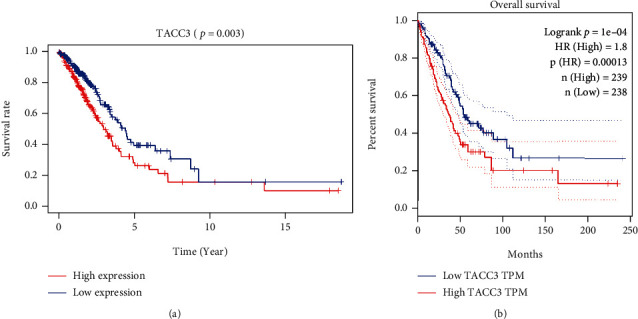
Analysis of survival outcomes. (a) *TACC3* expression and OS in LUAD patients in the TCGA cohort. The reduction of *TACC3* expression is linked to a good prognosis. (b) Survival analysis outcomes from the GEPIA database for validation.

As illustrated in Figure 6(a), elevated expression of *TACC3* was considerably linked to dismal OS (*P* =0.003). The observed correlation was additionally substantiated in the GEPIA database (Figure 6(b), *P* = 0.00013). The univariate analysis illustrated that elevated *TACC3* expression was considerably liked with dismal OS (hazard ratio (HR): 1.022; 95% CI: 1.009-1.04; *P* = 0.02) (Table 2). The other clinical features linked to worse survival comprised lymph node, clinical stage, and tumor status. The multivariate analysis was premised on these variables. The multivariate Cox analysis illustrated that elevated *TACC3* expression endured as an independent risk factor for OS having an HR of 1.027 (95% CI: 1.009–1.04, *P* = 0.002), as well as age and clinical stage among LUAD patients (Table 3).

### 3.5. Association between *TACC3* Expression and Composition of TIICs

**Figure 7:**
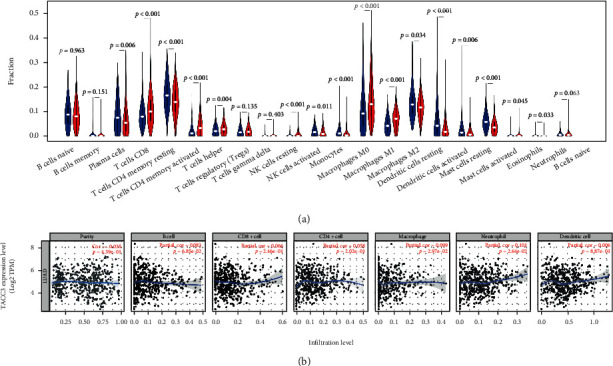
The outcomes of the relative proportions of TIIC that were extracted utilizing the CIBERSORT algorithm. (a) The ratio of 22 immune cells in LUAD tissues in the *TACC3* low and high expression cohorts. (b) The scatterplots illustrating the purity-corrected Spearman's rho value and *P* value of the correlation between *TACC3* and TIIC levels.

We aimed to assess the link between the expression of *TACC3* and various constituents of immune infiltrating cells in the LUAD TME. Premised on the median TACC3 expression value, we accessed 438 LUAD tumor tissues from the TCGA and categorized them into low and high expression cohorts. An aggregate of 219 low expression cohorts and 219 high expression cohorts were consistent with the criteria established for screening. Downloading of the gene expression profiles was done via the extensively recognized computation platform (CIBERSORT) to deduce the variation in the percentage of 22 immune cells between the two cohorts. The outcomes from the 22 immune cell subtypes are illustrated in Figure 7(a).

Results illustrated that immune cells which have a considerable association with *TACC3* expression included plasma cells (*P* = 0.006), CD4^+^ memory resting T cells (*P* < 0.001), CD4^+^ memory activated T cells (*P* < 0.001), CD8^+^ T cells (*P* < 0.001), follicular helper T cells (*P* = 0.004), NK resting cells (*P* < 0.001), NK activated cells (*P* = 0.011), monocytes (*P* < 0.001), M0 macrophages (*P* < 0.001), M1 macrophages (*P* < 0.001), M2 macrophages (*P* = 0.034), activated dendritic cells (*P* = 0.006), resting dendritic cells (*P* < 0.001), activated mast cells (*P* = 0.045), resting mast cells (*P* < 0.001), and eosinophils (*P* = 0.033). The results did not indicate any statistically significant intergroup variations in the infiltrates from other immune cells. The outcomes from the CIBERSORT algorithm illustrated that the percentages of follicular helper T cells, CD8^+^ T cells, CD4^+^ memory activated T cells, plasma cells, resting NK cells, activated NK cells, M0 macrophages, M1 macrophages, and M2 macrophages were considerably elevated in LUAD tumor tissues with an increased TACC3 expression. Moreover, the ratios of CD4 memory resting T cells, monocytes, resting dendritic cells, activated dendritic cells, M2 macrophages, resting mast cells, activated mast cells, and eosinophils were considerably elevated in the low expression cohort.

To validate these findings, “gene” panel in TIMER was employed to assess the link between immune infiltrating levels and *TACC3* (Figure 7(b)). The outcomes illustrated that *TACC3* had a positive association with the infiltrating levels of dendritic cell (*r* = 0.006, *P* = 8.87e − 01), neutrophils (*r* = 0.101, *P* = 2.64e − 02), and CD4^+^ T cell (*r* = 0.058, *P* = 2.02e − 01), but negatively correlated with tumor purity in LUAD (*r* = −0.035, *P* = 4.39e − 01), infiltrating levels of macrophages (*r* = −0.009, *P* = 2.64e − 02), CD8^+^ T cells (*r* = −0.066, *P* = 1.46e − 01), and B cells (*r* = −0.083, *P* = 6.85e − 02).

To further confirm these findings, we additionally assessed the connection between *TACC3* and distinct subtypes of infiltrating immune cells. The outcomes illustrated that the gene markers influenced by *TACC3* expression comprise CD40L of CD4^+^ T cell, CD8A and CD8B of CD8^+^ T cell, CD79A of B cell, PD-1(CD279), CTLA-4, LAG3, and TIGIT of T cell exhaustion, TBX21, and IFNG of Th1, IL21 of TFH, and FOXP3 of Treg (Table 4). We utilized the Spearman correlation coefficient in evaluating the correlation coefficient. The outcomes of *TACC3* and CD4^+^ T cell, CD8^+^ T cell, and NK cells markers showed a similarity when compared to those obtained from the CIBERSORT. The association between *TACC3* and different immune cell surface markers illustrated that it could serve as an immune target and candidly interrelate with immune cells. Past research reports have illustrated that viruses and proteins can identify immune-stimulating signals through the interaction with immune cell surface markers. The signals were conveyed via T cell cross-excitation and antigen cross-presentation to kill the tumor cells [26, 27]. Hence, these results illustrated that *TACC3* could considerably influence the abundance of NK cells and T cells. Nevertheless, further research is warranted to examine whether *TACC3* is a critical factor for B cell immune infiltration.

### 3.6. TACC3 Protein Interaction Network and Functional Enrichment Analysis

**Figure 8:**
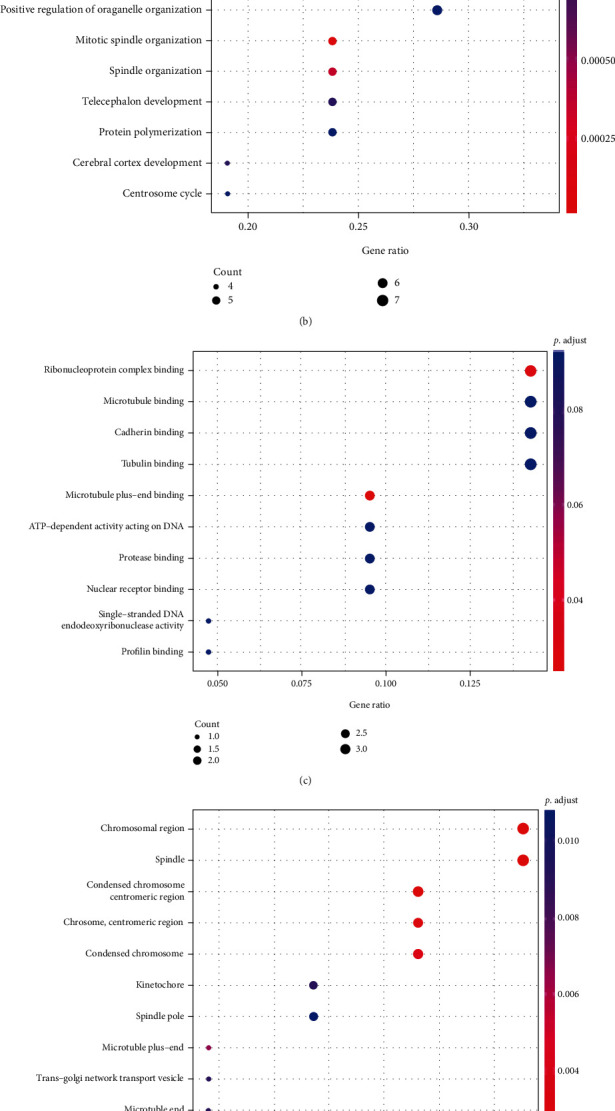
(a) A PPI network for TACC3. (b) The biological processes (BP) enrichment analysis. (c) The cellular component (CC) enrichment analysis. (d) The molecular function (MF) enrichment analysis. (e) The KEGG enrichment analysis.

The TACC3 protein interaction network was constructed using the GeneMANIA online tool to explore the role of TACC3 in the occurrence and development of cancer. As can be seen from the figure, the proteins that physically interact with TACC3 include CLIP4, EIF3C, and PANK2 (Figure 8(a)). The protein function enrichment analysis in the network graph is shown in the figure (Figures 8(b)–8(d)). The GO enrichment results showed that the biological processes involved in these proteins were organelle fission, microtubule cytoskeleton organization involved in mitosis, nuclear division, etc. (Figure 8(b)). The cellular components involved mainly include chromosomal region and condensed chromosome and spindle (Figure 8(c)). The molecular functions involved include ribonucleoprotein complex binding, cadherin binding, and tubulin binding (Figure 8(d)). According to the KEGG analysis results, the main enriched pathways are nonhomologous end-joining, RNA transport, pantothenate and CoA biosynthesis, homologous recombination, and nucleotide excision repair and other related pathways (Figure 8(e)).

### 3.7. Single-Cell Analysis of TACC3 in Cancers

**Figure 9:**
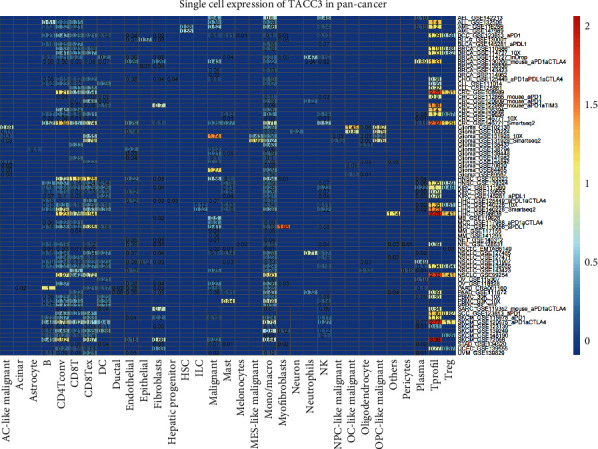
Summary of PDIA3 expression of 33 cell types in 79 single cell datasets.

To understand the main cell types that express the TACC3 in cancer microenvironments, we performed the single-cell analysis of TACC3 in 78 single-cell datasets of cancer samples. The heatmap depicted in Figure 9 represents the expression levels of TACC3 of 33 cell types (including immune cells, stromal cells, malignant cells, and functional cells) in 78 datasets using the TISCH web tool. The results indicated that TACC3 was mainly expressed in the immune cells (especially tprolif cells) and malignant cells.

### 3.8. Gene Set Enrichment Analysis

**Figure 10:**
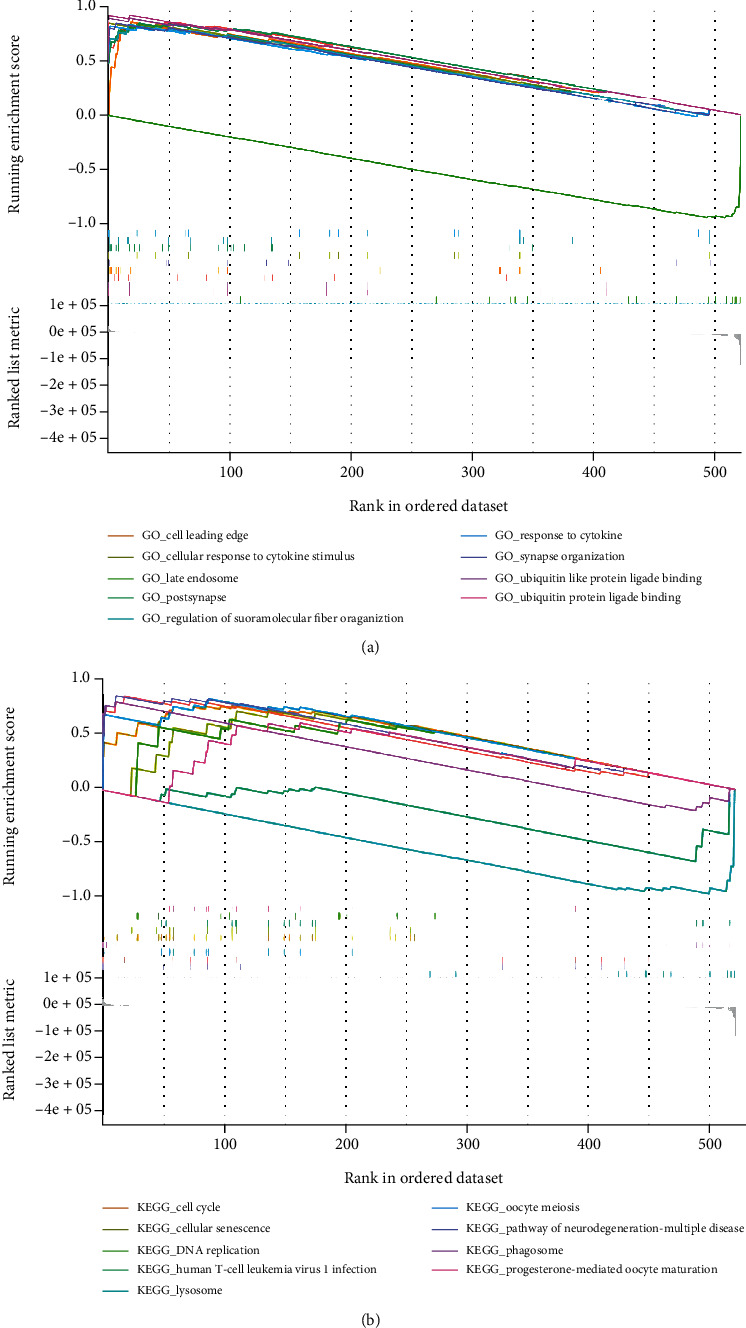
GSEA with KEGG pathway and GO term. (a) GO term analysis illustrated the most 5 positively linked pathways and 5 negatively linked pathways. (b) KEGG pathways illustrated the most 5 positively linked pathways and 5 negatively linked pathways.

To expound on the biological role of *TACC3* expression, we executed GSEA utilizing the KEGG pathway and GO terms. The assessment criterion for the outcomes was normalized enrichment score |NES| > 1 (*P* < 0.05). Subsequently, we chose the 10 most pertinent signal pathways premised on the absolute value of the NES. The GO terms illustrated that ubiquitin protein ligase binding, ubiquitin-like protein ligase binding, axon, cell leading edge, synapse organization, cellular response to cytokine stimulus, postsynapse, regulation of supramolecular fiber organization, and response to cytokine were most positively correlated with *TACC3* expression. The late endosome exhibited a considerable negative relationship with *TACC3* expression (Figure 10(a)). GSEA analysis selects the top 10 pathways according to the enrichment score. Among them, 8 KEGG pathways illustrated positively correlated with TACC3 expression: pathways of neurodegeneration-multiple diseases, Alzheimer disease, oocyte meiosis, phagosome, cell cycle, cellular senescence, DNA replication, and progesterone-mediated oocyte maturation. The 2 most negatively related categories included lysosome and human T cell leukemia virus 1 infection (Figure 10(b)). The all-inclusive analysis of the outcomes above illustrated that the *TACC3* gene stimulated cell cycle, DNA replication, progesterone-mediated oocyte maturation and pathways of neurodegeneration multiple diseases, response to cytokine, and human T cell leukemia virus 1 infections.

### 3.9. Pancancer Analysis of the Correlation between the TACC3 Expression and TMB and MSI

**Figure 11:**
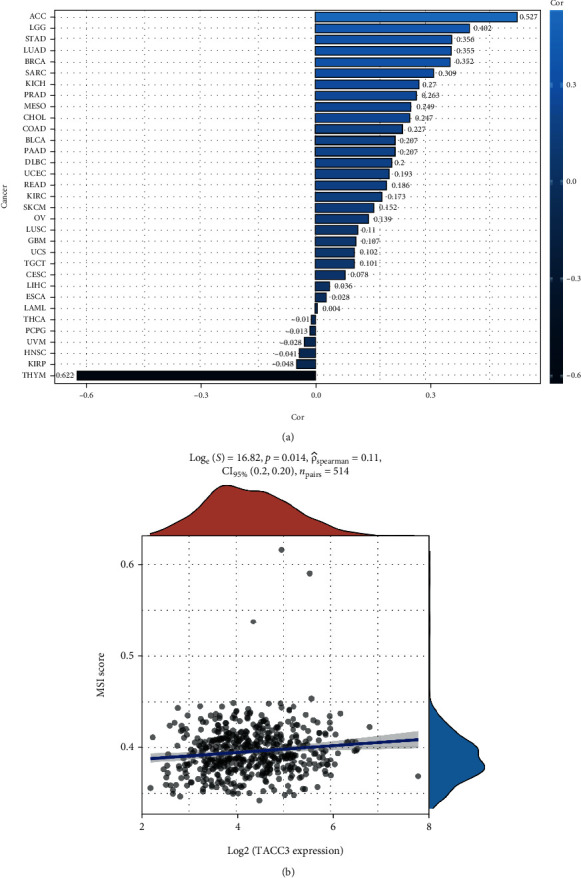
Correlation TACC3 with TMB and MSI. (a) Correlations between TACC3 expression and tumor mutation burden in pancancer. (b) Correlations between TACC3 expression and microsatellite instability in LUAD.

To explore the role of TACC3 in tumor microenvironment (TME) immune mechanisms and immune responses, we analyzed the correlation between TACC3 expression and TMB and MSI. TMB and MSI in the tumor microenvironment are related to antitumor immunity and can predict the efficacy of tumor immunotherapy. Our results showed that TACC3 expression was positively correlated with TMB in ACC, LGG, STAD, LUAD, BRCA, and SARC and negatively correlated in THYM (Figure 11(a)). In LUAD, TACC3 expression trended positively with MSI (Figure 11(b)).

### 3.10. TACC3 Expression Is Related to Immune Checkpoint (ICP) Genes in Human Cancers

**Figure 12:**
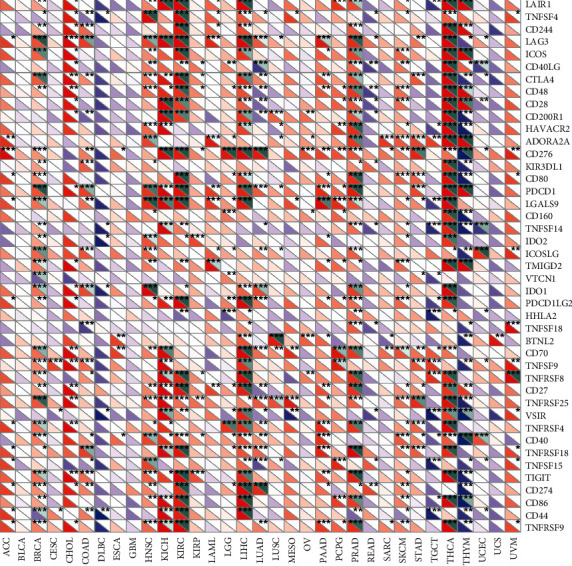
The relationship between TACC3 expression and pancancer immune checkpoint genes.

Studies have demonstrated that immune checkpoint (ICP) genes have a large impact on immune cell infiltration and immunotherapy. Subsequently, we explored the association between TACC3 expression and ICP genes in human cancers to explore the potential of TACC3 in immunotherapy. Among the 47 ICP genes, multiple cancer types closely associated with TACC3 expression were found, such as KICH, KIRC, THCA, THYM, CHOL, DLBC, ACC, and UVM (Figure 12). Except for THYM and DLBC, TACC3 expression was positively correlated with immune checkpoint genes, especially in LIHC, where 26 out of 47 immune checkpoint genes were associated with TACC3 expression. In LUAD, however, TACC3 was only positively associated with two immune checkpoint genes, CD274 and CD276. This suggests that TACC3 may coordinate the activities of these ICP genes in different signal transduction pathways and may be an ideal immunotherapy target.

### 3.11. TACC3 and Drug Response

**Figure 13:**
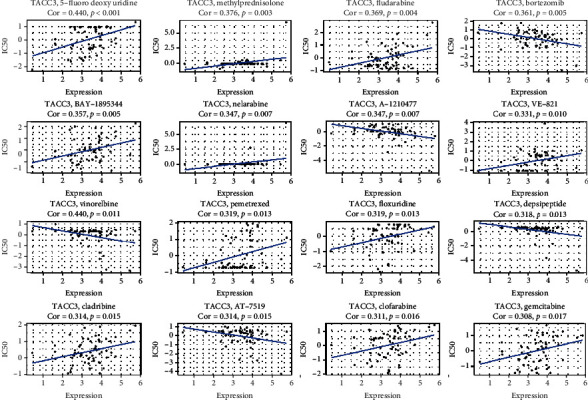
An illustration of the relationship between TACC3 expression and expected medication response.

TACC3 expression was positively connected with drug response in patients treated with 5-fluorodeoxyuridine-10, methylprednisolone, fludarabine, BAY-1895344, nelarabine, VE-821, pemetrexed, floxuridine, cladribine, clofarabine, and gemcitabine. Additionally, there is negative connection between TACC3 expression and the anticancer drugs. These anticancer drugs mainly are bortezomib, A-1210477, vinorelbine, depsipeptide, and AT-7519. An illustration of the relationship between TACC3 expression and expected medication response can be found in Figure 13.

## 4. Discussion

Over the last 3 decades, LUAD treatment has seen a dramatic evolution, particularly with the effectiveness of immunotherapy [28]. This illuminates the critical function of T cell immunoglobulin mucin (TIM) in the development and progression of LUAD. As such, it is of great importance to detect the immune-related biomarkers and clarify the fundamental molecular mechanism of LUAD to enhance the prognosis of patients and offer guidance regarding the development of effectual therapeutic interventions. This research was centered on TACC3, which is a well-recognized member of the TACC family and a centrosome and microtubule-associated protein [29]. Whereas the function of *TACC3* is yet to be comprehensively clarified, cumulative proof from research reports indicates that *TACC3* is necessary for attachment of kinetochore-microtubule, assembly of centrosome dependent microtubule, and the alignment of spindle-dependent chromosome in the process of mitosis [30, 31]. Besides its role in mitosis, *TACC3* participates in the control of cell growth, differentiation, and transcriptional regulation [32, 33]. In addition, research reports have discovered that *TACC3* is an imperative prognostic biomarker for kidney renal clear cell carcinoma and is correlated with immune cell infiltration as well as the depletion of T cells [34]. Owing to the limited current knowledge regarding the function of *TACC3* in cancers, we intended to scrutinize its biological roles in LUAD and divulge its related regulatory pathways through conducting an all-inclusive analysis of open-access datasets.

In this research, we discovered via the online dataset GEPIA that there is a noteworthy relationship between *TACC3* and the LUAD prognosis. The outcomes from this research also illustrated that *TACC3* expression is different in LUAD tissues while contrasted with normal lung tissues. The elevated *TACC3* expression forecasts a dismal prognosis. Furthermore, the elevated *TACC3* expression influenced the clinical factors of T classification, clinical stage, and age. This research also discovered that various subsets of immune markers and the levels of many immune cell infiltrations are associated with the differential *TACC3* expression in LUAD. It was further illustrated that *TACC3* might influence tumor immunogenicity and become a possible biomarker for forecasting the prognosis of tumors. To additionally examine the probable mechanism of the expression of *TACC3* in LUAD and its link to clinical features, we accessed up-to-date data from the public dataset, the TCGA. The association between LUAD and *TACC3* was ascertained via the Cox regression analysis and GEPIA. The CIBERSORT was utilized to identify the relative ratios of various TIICs in the TME to assess the link between the expression of *TACC3* and tumor immune infiltration in order to establish its effect on the prognosis of LUAD patients. These outcomes might be useful in providing a basis for further examination of the application value of *TACC3* in LUAD and disclose the probable relationship and probable mechanism of *TACC3* expression and immune interplay in the TME. Also, *TACC3* could have a possible application value in the immunosuppression resulting from the TME. When considered as a novel target for the regulation, it could have the capacity to be utilized in conjunction with immune checkpoints to clarify the extent of immunotherapy benefits to the population and enhance the impacts of immunotherapy. Thus, *TACC3* could be a novel marker for assessing the immune infiltration and prognosis of LUAD patients.

Life activities are usually coregulated by various genes. As a prognostic factor of LUAD, TACC3 can be more clearly understood through PPI network research. The study found that TACC3 involved a total of 21 protein molecules in the PPI network, and GO and KEGG analysis showed that these protein sets were significantly enriched in some pathways such as homologous recombination and nucleotide excision repair. It is speculated that in LUAD patients, the loss of TACC3 function will cause DNA repair dysfunction.

TISCH is a scRNA-seq database focused on the tumor microenvironment (TME). TISCH provides detailed cell-type annotation at the single-cell level, enabling the exploration of TMEs across different cancer types. Previously, we identified the key role of TACC3 in the development of LUAD, and we also wanted to explore the expression level of TACC3 in different cell types. The results of TISCH database analysis showed that TACC3 is mainly expressed in the immune cells (especially tprolif cells) and malignant cells. The correlation analysis with immune check genes found that in LUAD, TACC3 expression was positively correlated with CD274 and CD276. Based on these results, it is speculated that TACC3 plays a role in multiple immune gene signaling pathways.

Premised on the regression analysis, we discovered that *TACC3* serves as an independent factor in the prognosis of LUAD. In this research, a noteworthy limitation observed is that the *TACC3* expression in LUAD is correlated with the immune infiltration level. The CIBERSORT analysis indicated that the *TACC3* expression had a considerable influence on the infiltration levels of NK cells and T cells in the LUAD TME. The correlation between TACC3 and tumor mutational burden (TMB) and microsatellite instability (MSI) also demonstrated that TACC3 is closely related to TME in human cancers. At the same time, we compiled a list of more than 40 common immune checkpoint genes and estimated the correlation between their expression and TACC3 expression, and we believe that immunotherapy can be estimated by detecting the expression level of CDCA4 effect in the future. Correspondingly, the link between the expression of genetic markers by various immune cells and *TACC3* denotes that *TACC3* performs a regulatory function in the immune microenvironment of LUAD. As per the results from the CIBERSORT algorithm, it was discovered that the proportions of activated NK cells, resting NK cells, follicular helper T cells, CD4 memory activated T cells, CD8^+^ T cells, plasma cells, M0 macrophages, M1 macrophages, and M2 macrophages were considerably elevated in LUAD tumor tissues with an increased TACC3 expression. Additionally, the ratios of CD4 memory resting T cells, resting mast cells, activated mast cells, activated dendritic cells, resting dendritic cells, monocytes, M2 macrophages, and eosinophils were considerably elevated in the low expression cohort.

Subsequently, we employed the “Correlation” module in GEPIA to validate these findings. The relationship between *TACC3* and the expression of surface markers in immune cells was fundamentally similar. Such a relationship might indicate a probable mechanism under which *TACC3* participates in the regulation of the activities of immune cells in LUAD. Next, we assessed an additional imperative aspect, since the *TACC3* expression is associated with numerous recognized pathways in the immune responses and cancer processes. The *TACC3* gene stimulated cell cycle and homologous recombination and inhibited nucleotide excision repair and lamellar body, aldosterone-regulated sodium reabsorption, and primary bile acid biosynthesis. The ratio of immune cell infiltration has a crucial function in the tumor cell survival, metastasis, and resistance to treatment [35]. The evolving immunotherapies which include PD-L1, PD-1, and CTLA4 suppressors have led to extremely effectual antitumor impacts in cancer therapies [36, 37]. Nevertheless, PD-L1 is incapable of having a decisive function in therapies that involves chemoimmunotherapy [38]. As such, there is a pressing need to discover more immune mechanisms or immune targets to aid in the treatment of LUAD. Numerous research reports have illustrated that the expression levels of the majority of genes are linked to dismal tumor prognosis and the proportion of immune cell infiltration to tumor cells.

In a wide range of cancers, it has been suggested that CD8^+^ cytotoxic T cells (CTLs) act as the main effectors in the destruction of tumor cells. Its elevated infiltration levels have been found to be positively linked to a good clinical prognosis [39]. During cancer progression, the CTLs experience depletion and dysfunction owing to immunosuppression and immune-related tolerance and within TME, with all favoring the adaptive immune-resistance [40]. On the other hand, the CD8^+^ T cell priming has been found to be directed fundamentally as a substantiation function between the cells of innate immunity including natural killer (NK) cells and dendritic cells (DCs) with CD4^+^ T cells within the adaptive immunity. After activation, the effector CTLs tend to infiltrate the invading site or the core of the tumor (commonly referred to as the infiltrated-inflamed (I-I) TME) and perform the essential function of killing the cancer cells. While greater attention has been given to the function of CTLs in tumor protection, it has been established that CD4^+^ helper T cells also have a crucial function in cancer immune responses in both patients and animal models [41]. Particularly, CD4^+^ Treg cells have been found to possess a great immunosuppressive role and enable the progression of the tumor through inhibiting effectual antitumor immunity [42]. It has also been illustrated that CD4^+^ CTLA-4^+^ T cells in circulation are considerably elevated in patients with advanced cancer stages [43]. The existence of T cells within the tumor parenchyma is a common feature of immunoinvasive tumors, illustrating the antitumor impact of antitumor antigen T cells within the immunosuppressive microenvironment [44]. Cumulative proof from research reports illustrates that defective T cells infiltrate into tumors, which denotes one of the common mechanisms for medication resistance mechanisms to immunotherapy [45]. The natural killer (NK) cells have been identified as the innate cytotoxic lymphocytes which participate in the surveillance as well as the purging of cancer [46]. The NK cells offer the first line of defense which could orchestrate and bridge “downstream” and innate adaptive immune responses, thus making them an ideal foundation on which new cancer treatments could be based [47]. Amounting evidence from clinical studies and scientific reports has illustrated auspicious antitumor impacts when utilizing NK cell-based immunotherapy [48]. In the TME, the suppression of immune effortlessly takes place as a result of the interplay between immune cells, and this has been found to influence immune monitoring. An ability to assess immune cell infiltration into tumors and the receptor repertoires of the infiltrating immune effector cells would improve predictions of clinical response and make it possible to design personalized immunotherapies based on a better stratification of patients with cancer. The factors critical to the success of future treatments thus include the development of ways of increasing tumor infiltration and rescuing the function of immune effector cells.

To surmise, this research discovered that the expression of *TACC3* could influence the constituent proportion of NK cells and T cells in the immune microenvironment of LUAD tumor tissues, thus incidentally influencing tumor progression and controlling immune surveillance. As of recent times, there have been numerous debates concerning the advantages of immune checkpoint suppressors in therapeutic interventions for tumors. The immunocheckpoints including CTL-4 or PD-1 might not be the only indicators in immunotherapy. These checkpoints could be utilized as auxiliary indicators to differentiate the advantageous and disadvantageous cohorts of immunotherapy and to enhance the precision and efficacy of immune checkpoint suppressors in tumor therapeutics. Future prospective studies focusing on TACC3 expression and tumor immune milieu will help provide conclusive answers to develop immune-based anticancer therapies.

## 5. Conclusions

A dismal prognosis in LUAD patients was contingent on the elevated expression of *TACC3*. *TACC3* might be regarded as an early diagnostic as well as an independent prognostic factor for patients with LUAD. Furthermore, elevated *TACC3* expression correlates with high immune infiltration levels in CD4^+^ T cells, CD8^+^ T cells, and most of the functional NK cells. Therefore, *TACC3* most probably has a critical impact on immune infiltration and might act as a probable prognostic biomarker of LUAD.

This research offered a novel probable marker for clinical forecasting of the survival of patients and provides an elementary foundation supporting the development of an innovative immunotherapy target. This could also offer a novel way for advancing personalized immunotherapy of LUAD and for the benefit of a greater number of patients. Nevertheless, there exists few limitations in this study. The research mainly relied on data mined from existing databases. Whereas we made efforts to verify the data utilizing the IHC technique, functional experiments in this area still lacking. In future research, our emphasis will be on improving these areas.

## Figures and Tables

**Table 1:** Logistic regression of *TACC3* expression and clinical pathological characteristics.

Clinical characteristics	Odds ratio for high *TACC3* expression	95% CI	*P* value
Age			
<65 vs. >65	1.8305	1.263-2.662	0.00146
Gender			
Male vs. female	1.344	0.936-1.931	0.108
Clinical stage			
II vs. I	1.912	1.216-3.028	0.0052
III vs. I	1.5905	0.956-2.659	0.074
IV vs. I	1.647	0.722-3.847	0.236
Tumor status			
T2 vs. T1	1.538	1.033-2.297	0.034
T3 vs. T1	1.242	0.621-2.478	0.536
T4 vs. T1	1.449	0.554-3.836	0.44
Lymph node			
N1 vs. N0	1.795	1.11.3-2.931	0.017
N2 vs. N0	1.571	0.9305-2.675	0.09
Distant metastasis			
M1 vs. M0	1.435	0.623-3.418	0.399

**Table 2:** Relationship between*TACC3* expression and clinicopathological parameters of LUAD patients.

Clinicopathological parameters	TACC3 expression			*P* value
	High	Low	Negative	
N	50	24	7	
Age				
Mean (SD)	61.48(9.52)	62.50 (6.91)	65.00 (6.43)	0.578
Sex (%)				
Female	21(42.0)	12 (50.0)	4 (57.1)	0.662
Male	29 (58.0)	12 (50.0)	3 (42.9)	
T (%)				
T1	24 (48.0)	16 (66.7)	6 (85.7)	0.291
T2	23 (46.0)	7 (29.2)	1 (14.3)	
T3	3 (6.0)	1 (4.2)	0 (0.0)	
N (%)				
N0	26 (52.0)	17 (70.8)	6 (85.7)	0.061
N1	11 (22.0)	0 (0.0)	1 (14.3)	
N2	13 (26.0)	7 (29.2)	0 (0.0)	
M (%)				
M0	50 (100.0)	24 (100.0)	7 (100.0)	NA
Clinical stage (%)				
I	24 (48.0)	16 (66.7)	6 (85.7)	0.097
II	12 (24.0)	1 (4.2)	1 (14.3)	
III	14 (28.0)	7 (29.2)	0 (0.0)	
Smoking history (%)				
Yes	32 (64.0)	15 (62.5)	6 (85.7)	0.494
No	18 (36.0)	9 (37.5)	1 (14.3)	
Survival status (%)				
Yes	28 (56.0)	9 (37.5)	0 (0.0)	0.013
No	22 (44.0)	15 (62.5)	7 (100.0)	

**Table 3:** Univariate and multivariate analysis of the relationship between *TACC3* expression and overall survival among LUAD patients.

Parameter	Univariate analysis			Multivariate analysis		
	HR	95% CI	*P* value	HR	95% CI	*P* value
Age	1.012414	0.99575-1.029357	0.145103	1.018965	1.00255-1.03565	0.023375
Gender	1.08451	0.78743-1.493671	0.619379	0.971737	0.699646-1.349644	0.864182
Stage	1.655573	1.426762-1.921079	3.07E-11	1.478029	1.189063-1.83722	0.000431
T	1.535725	1.25942-1.872647	2.24E-05	1.169061	0.946446-1.444039	0.147261
N	1.699193	1.419914-2.033402	7.17E-09	1.204262	0.944326-1.535749	0.134076
TACC3	1.022942	1.005876-1.040297	0.008228	1.027566	1.009751-1.045695	0.002307

**Table 4:** Spearman correlation analysis between *TACC3* and markers of immune cells in patients with LUAD and normal tissue.

Description	Gene markers	LUAD			
		Tumor		Normal	
		*R*	*P*	*R*	*P*
CD4^+^ T cell	CD4	0.062	0.083	0.0015	0.4
	CD40L	-0.27	1.2e−09	0.41	0.0014
	CXCR4	0.21	0.057	0.39	0.0024
CD8^+^ T cell	CD8A	0.29	0.024	0.12	0.011
	CD8B	0.32	0.014	0.14	0.0026
T cell (general)	CD2	−0.0055	0.9	0.30	0.012
	CD3E	0.023	0.62	0.51	3.7e−05
B cell	CD19	−0.024	0.59	0.4	0.0015
	CD79A	-0.12	0.0091	0.38	0.0029
T cell exhaustion	PD-1(CD279)	0.27	1.1e−09	0.58	1.8e−06
	CTLA-4	0.17	0.00016	0.39	0.0022
	LAG3	0.33	4.1e−14	0.31	0.0094
	TIM3	0.043	0.35	0.2	0.13
	GZMB	0.37	3.2e−17	0.16	0.18
	SLAMF4	−0.021	0.65	−0.026	0.65
	PD-L1	0.26	4.2e−09	0.21	0.1
	CD96	−0.022	0.63	−0.022	0.62
	IDO1	0.2	1.1e−05	0.094	0.48
	KDR	−0.073	0.11	0.097	0.47
	PD-L2	0.097	0.033	0.059	0.66
	TGFBR1	0.042	0.36	0.23	0.08
	TIGIT	0.17	0.00016	0.62	1.8e−07
Natural killer cell	KIR2DL1	0.069	0.13	0.17	0.19
	KIR2DL3	0.17	0.00019	0.15	0.24
	KIR2DL4	0.38	2.8e−18	0.083	0.53
	KIR3DL1	0.15	0.0014	0.21	0.12
	KIR3DL2	0.12	0.0084	0.23	0.079
	KIR3DL3	0.18	8.5e−05	0.027	0.84
	CD56	−0.04	0.38	0.06	0.7
Neutrophils	CD66b	-0.31	2.9e−12	0.085	0.52
	CD11b	0.0065	0.89	0.44	0.00054
	CCR7	−0.085	0.063	0.58	1.5e−06
Th1	TBX21	0.15	0.0013	0.44	0.00054
	STAT4	0.078	0.087	0.53	1.9e−05
	STAT1	0.38	4.4e−18	0.24	0.073
	IFNG	0.26	6.2e−09	0.27	0.037
Th2	STAT6	−0.06	0.19	0.52	2.5e−05
	STAT5A	0.059	0.19	0.64	4e−08
TFH	BCL6	−0.019	0.67	0.16	0.23
	IL21	0.14	0.0017	0.36	0.0052
Th17	STAT3	−0.04	0.38	0.24	0.071
	IL17A	0.088	0.053	0.00097	0.99
Treg	FOXP3	0.18	5.1e−05	0.55	8e−06
	STAT5B	0.065	0.15	0.38	0.0033
	TGFB1	0.022	0.62	0.57	1.9e−06
Monocyte	B7-2	0.01	0.82	0.32	0.012
	CSF1R	0.017	0.71	0.61	2.7e−07

Note: R: *P* value of Spearman's correlation. Tumor: correlation analysis in LUAD tumor tissue of TCGA. Normal: correlation analysis in LUAD normal tissue of TCGA.

## Data Availability

The datasets used during the present study are available from TCGA (https://portal.gdc.cancer.gov/repository) database or from the corresponding author upon reasonable request.
